# Embolization of Deep Femoral Artery Aneurysm with a Ligated Proximal Artery Using the Direct Percutaneous Puncture Technique

**DOI:** 10.3400/avd.cr.22-00043

**Published:** 2022-12-25

**Authors:** Kohei Hamamoto, Takao Nonaka, Koichi Tamai, Emiko Chiba, Noriko Oyama-Manabe, Yohsuke Suyama, Sadahiro Watanabe, Eiko Hyoe, Hiroshi Shinmoto

**Affiliations:** 1 Department of Radiology, National Defense Medical College, Tokorozawa, Saitama, Japan; 2 Department of Radiology, Jichi Medical University Saitama Medical Center, Saitama, Saitama, Japan; 3 Department of Cardiovascular Surgery, Jichi Medical University Saitama Medical Center, Saitama, Saitama, Japan; 4 Department of Radiology, National Center Hospital, National Center of Neurology and Psychiatry, Kodaira, Tokyo, Japan

**Keywords:** deep femoral artery aneurysm, direct puncture, embolization

## Abstract

We report a case of a deep femoral artery aneurysm with a ligated proximal artery that was successfully managed with endovascular therapy. An 84-year-old male was referred to our institute with a history of surgical resection of a left ruptured deep femoral artery aneurysm wherein another aneurysm was found on the peripheral side. Proximal artery ligation of the peripheral lesion was performed. The residual aneurysm had gradually enlarged after surgery, and contrast-enhanced computed tomography showed contrast effects in the aneurysm that extended to the distal artery. The aneurysm was successfully treated by direct percutaneous puncture embolization with *N*-butyl-cyanoacrylate.

## Introduction

A true deep femoral artery (DFA) aneurysm is an extremely rare condition.^1–3)^ Because enlarged DFA aneurysms have a high rupture rate, early treatment is crucial. Historically, DFA aneurysms have been managed with open surgical repair.^1–4)^ Nonetheless, in recent years, endovascular interventions such as coil embolization and stent graft placement have become attractive treatment alternatives as they are less invasive.^5–7)^ However, these endovascular treatments are challenging in cases having a lesion with a ligated proximal artery as access to the responsible vessels becomes complex.

Here, we report a case of a DFA aneurysm that was successfully treated by direct percutaneous puncture embolization with *N*-butyl-cyanoacrylate (NBCA).

## Case Report

**Figure 1:**
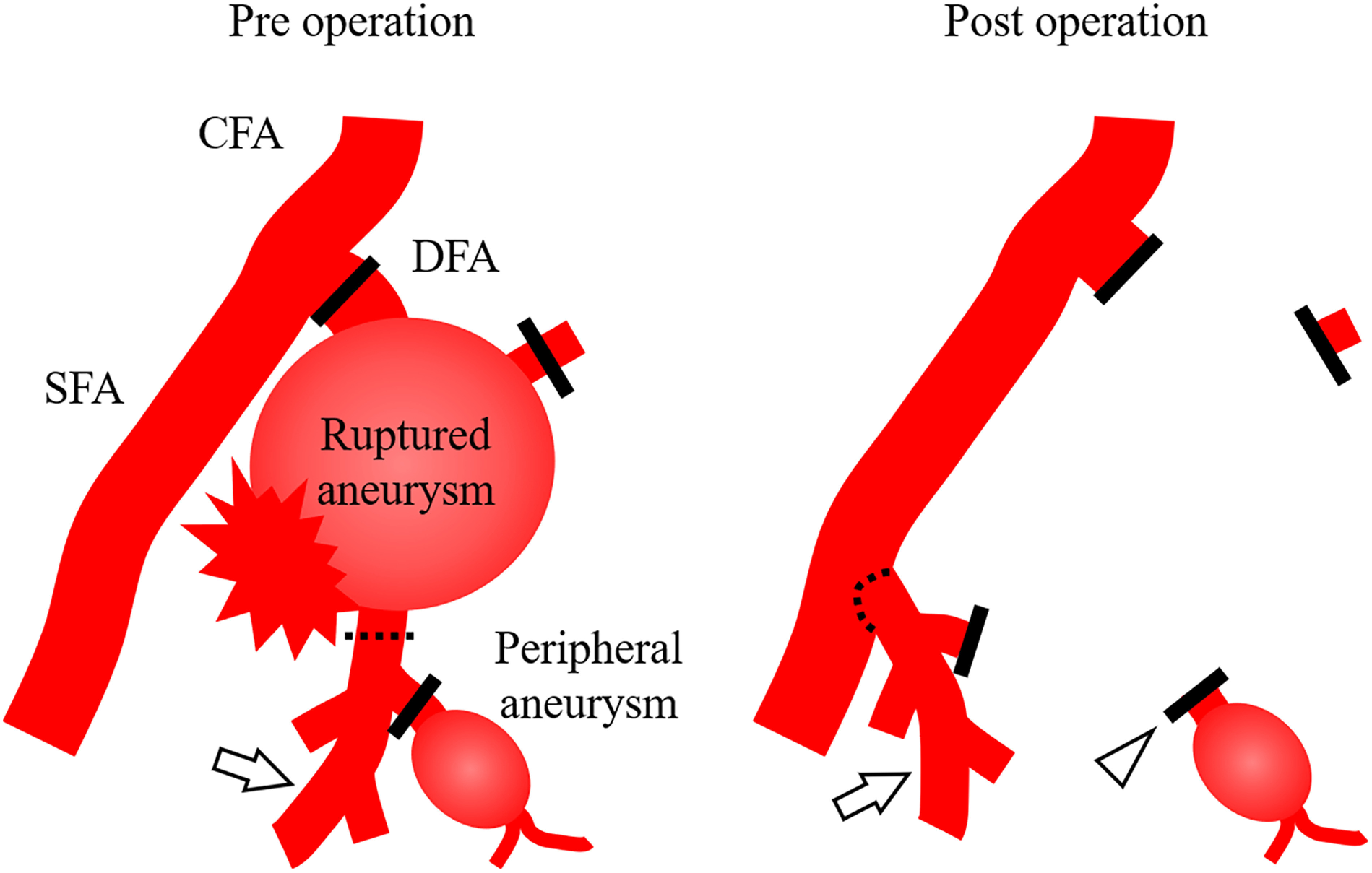
Fig. 1 Schema of the previous operation for ruptured deep femoral artery (DFA) aneurysm. The ruptured aneurysm is resected, and the main trunk of DFA is reconstructed. The proximal artery of the peripheral aneurysm is ligated (arrowhead). Solid lines indicate ligation; dashed lines indicate reconstruction.

CFA: common femoral artery; SFA: superficial femoral artery

**Figure 2:**
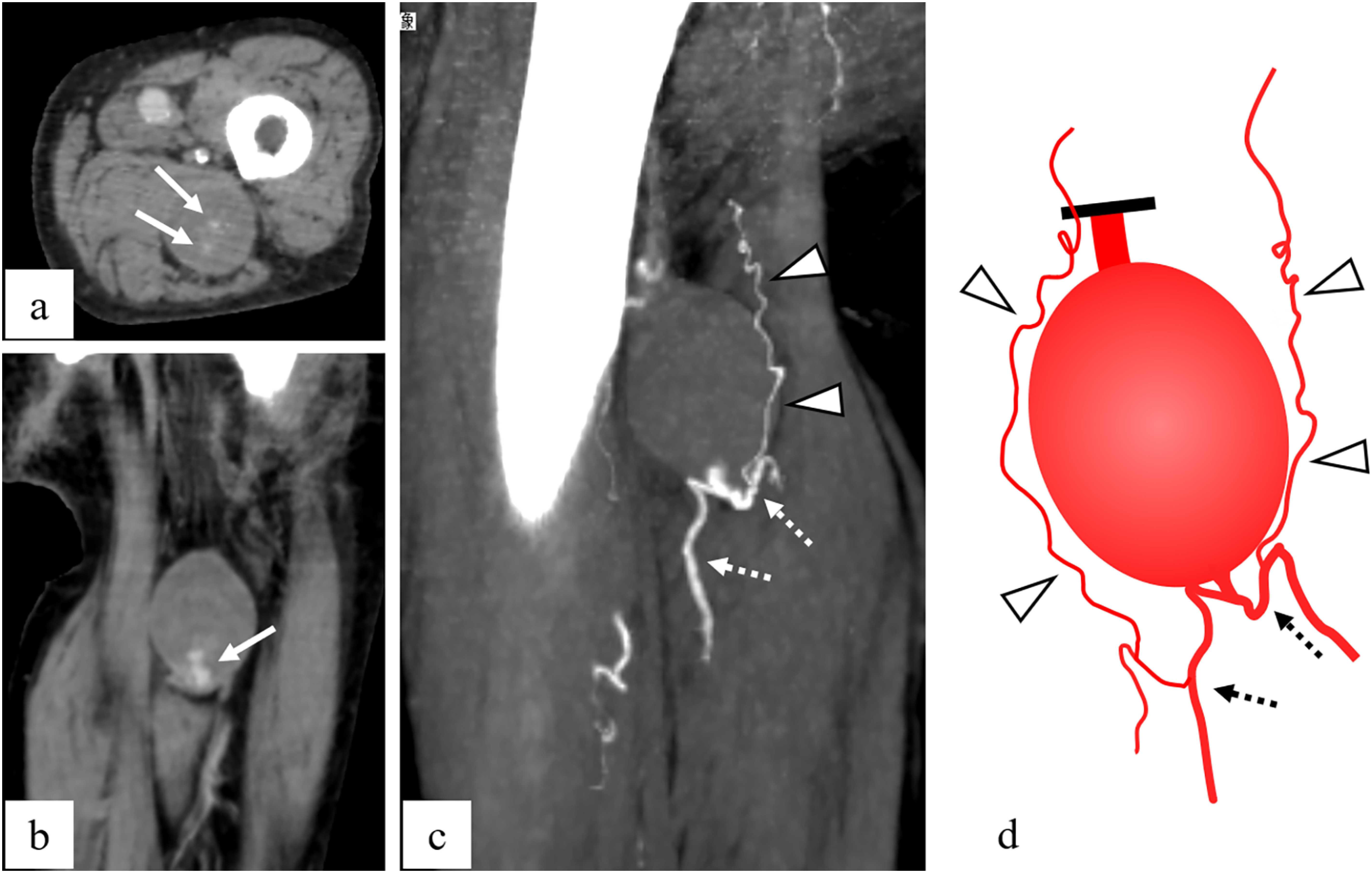
Fig. 2 Contrast-enhanced computed tomography (CECT) at 6 months after the previous operation. Contrast effects in the aneurysm (arrows in **a** and **b**) continuous with the tortuous distal arteries (dashed arrows in **c**) are noted. Arrowheads in **c** indicate a collateral vessel from the internal iliac artery. (**d**) Schema of the CECT image. The solid line indicates ligation; arrowheads and dashed arrows indicate collateral vessels and distal arteries and collateral vessels, respectively.

**Figure 3:**
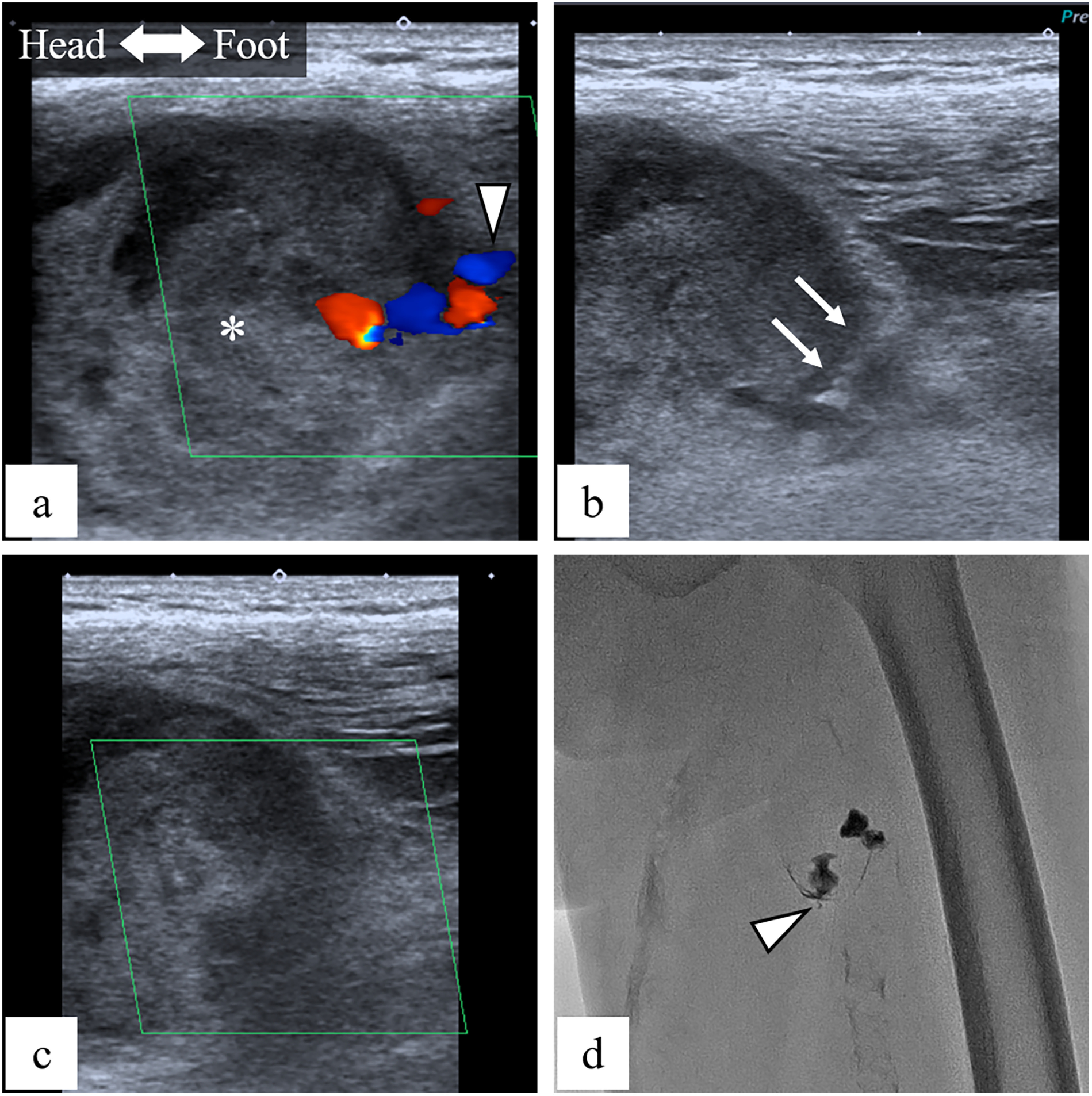
Fig. 3 Direct puncture percutaneous embolization. Color Doppler ultrasonography shows blood flow signals in the distal side of the aneurysm and the proximal portion of the distal artery (arrow) (**a**). A 21-G needle is introduced into the ostium of the distal artery; arrows indicate the puncture needle line (**b**). Color Doppler ultrasonography after the embolization shows that the blood signals have completely disappeared (**c**). The X-ray image after embolization shows deposition of Lipiodol in the distal side of the aneurysm and the proximal portion of the distal artery (arrowhead) (**d**).

An 84-year-old male with a left DFA aneurysm was referred to our institute. Eight months prior to this referral, he underwent emergent surgical resection of a ruptured DFA aneurysm (63 mm×55 mm×56 mm) on the left side, wherein another DFA aneurysm (32 mm×31 mm×40 mm) was found on the peripheral side. Nonetheless, only proximal artery ligation of the peripheral lesion was performed at that time for the following reasons: 1) the emergency nature of the surgery, 2) the peripheral lesion was relatively small, with a lower risk of rupture, and 3) the peripheral lesion was located in the deep layer in the thigh. Therefore, the resection was considered highly invasive (**Fig. 1**). The size of the residual aneurysm gradually increased during follow-up after surgery, and he was directed to the radiology department for endovascular treatment. He had a history of graft replacement for an abdominal aortic aneurysm, surgical resection of a right lateral circumflex femoral artery aneurysm, and Alzheimer’s disease with an eight-point score on Hasegawa’s Dementia Scale. Serum biochemical tests showed no obvious abnormalities, except for an elevated serum creatinine level (1.8 mg/dL). No abnormalities were detected on a complete blood count analysis and clotting test. He had no episode, family history, physical findings, or laboratory findings indicating systemic vascular disease or vasculitis. He had taken aspirin, clopidogrel, antihypertensive drugs, and donepezil. Computed tomography (CT) showed a 40 mm×38 mm×52 mm aneurysm in the middle of the left DFA. The proximal artery of this lesion was ligated. Contrast-enhanced CT revealed a contrast effect in the aneurysm that extended to the distal artery. The distal artery branched into multiple small tortuous branches immediately after emerging from the aneurysm, which were continuous with the small collateral vessels from the branches of the left internal iliac artery and reconstructed left DFA (**Fig. 2**). No aneurysms were detected at other sites. Ultrasonography (US) showed that the majority of the aneurysm was thrombolyzed, but blood flow signals were identified in the distal portion of the aneurysm. Vascular surgeons and radiologists discussed whether to treat this aneurysm surgically or endovascularly and decided that the latter was suitable as first-line treatment owing to his numerous comorbidities. The patient and his family consented to this treatment plan. On the basis of the preprocedural imaging findings, we determined that a transcatheter arterial embolization (TAE) with an antegrade or transcollateral approach was not feasible. Additionally, we needed a procedure that could be completed in a short time period as the patient was expected to have difficulty staying in the same position for a long time due to Alzheimer’s disease. Therefore, we planned to perform embolization by directly puncturing the distal orifice of the aneurysm. The patient was placed in a prone position and sedated with continuous dexmedetomidine administration. We first identified the blood flow signals within the aneurysm and distal artery using an ultrasound instrument (Aplio 500, Canon Medical Systems, Otawara, Japan) and a 7.5-MHz linear array probe (PLT-704SBT, Canon Medical Systems) (**Fig. 3a**). Then, a 21-G Chiba needle was introduced into the distal orifice of the aneurysm under US guidance (**Fig. 3b**), and we carefully injected 1.2 mL of NBCA (B. Braun, Melsungen, Germany)–Lipiodol (Lipiodol Ultra-Fluid; Guerbet, Roissy, France) mixture (1 : 2 ratio) in a stepwise fashion under ultrasound guidance until the color flow signals disappeared. Subsequently, a second puncture and injection of 1.5 mL of the NBCA–Lipiodol mixture (1 : 2 ratio) were performed because a faintly residual blood signal was detected in the aneurysm. After the second injection, the blood signals completely disappeared (**Fig. 3c**). A radiograph acquired after this procedure showed the presence of Lipiodol in the distal side of the aneurysm and the proximal part of the distal artery (**Fig. 3d**). The procedure was well tolerated by the patient, and it took approximately 40 min to complete. Follow-up US performed on the next day showed no evidence of recanalization in the aneurysm and hematoma. The patient was discharged 2 days after embolization without any complications. No blood signals were detected in the aneurysm on US performed 4 months after embolization. On unenhanced CT performed 16 months after embolization, Lipiodol was still present within the aneurysm and proximal portion of the distal artery. The size of the aneurysm reduced to 37 mm×30 mm×45 mm (preoperative CT, 40 mm×38 mm×52 mm). US performed at nearly the same time revealed no evidence of residual blood flow in the aneurysm, and blood flow in the distal arteries was preserved.

## Discussion

We utilized a novel approach for the treatment of complex DFA aneurysms by direct percutaneous puncture embolization. DFA aneurysms are rare forms of peripheral arterial aneurysms and have been commonly associated with other peripheral aneurysms and occlusive diseases.^1–3)^ Because there are no characteristic symptoms for DFA aneurysms at an early stage, they are often diagnosed as large lesions with a high rupture risk. Surgical repair plays an important role in the treatment of DFA aneurysms, particularly in the case of ruptured or enlarged lesions. Several treatment options, including simple ligation, revascularization of the distal DFA, and bypass grafting to the popliteal artery, have been reported.^1–4)^ In recent years, endovascular interventions such as TAE with metallic coils, stent graft placement, and thrombin injection have been used for the treatment of true or pseudo-DFA aneurysms, mainly in patients with a high risk for surgery or multiple comorbidities.^5–8)^ In our case, stent graft placement and TAE with an antegrade or transcollateral approach were not feasible because the proximal artery was ligated during the previous operation, making the collateral vessels too small to access. Additionally, we needed to preserve blood flow in the collateral vessels and distal arteries of the target aneurysm to avoid ischemia in the deep femoral region. Therefore, we decided to perform selective embolization of the distal aneurysmal orifice by a direct puncture to achieve complete embolization. A similar direct puncture was previously used in the treatment of various vascular diseases, such as embolization for a type II endoleak after an endovascular abdominal aortic aneurysm repair, and an internal iliac artery aneurysm.^9,10)^ To our knowledge, this is the first case of a DFA aneurysm that was successfully managed by endovascular treatment using a direct puncture.

The main advantages of using a direct puncture are the following: 1) it is less invasive and can be performed in a short time, 2) the target aneurysm and its feeding arteries can almost certainly be delineated, and 3) embolic materials can be easily placed in the target lesions. In particular, its less invasiveness and short procedural time could be useful for patients who cannot maintain the same position for a long time, as was in our case. Its disadvantages include the possibility of bleeding and aneurysm formation at the puncture site should the embolization remain incomplete. However, the application of NBCA on the aneurysm and confirmation of complete hemostasis in the puncture site by US might help in resolving this issue. Additionally, we performed the procedure under US guidance, which provided several advantages: real-time monitoring of blood flow in the target lesions during embolization was possible, and radiation exposure was reduced in comparison to TAE, which requires fluoroscopy.

In our case, other embolic materials, including metal coils, gelatin sponges, and thrombin, were also available. Coil embolization generally requires the insertion of a catheter, which was difficult in this case because there was no space for catheter insertion. Moreover, we did not use gelatin sponges or thrombin because the embolic effect of these materials is dependent on the patient’s blood coagulability and may lead to uncertain hemostasis in patients taking antiplatelet agents, as was in our case. NBCA is a liquid embolic material that does not depend on the patient’s blood coagulability. Thus, we determined NBCA to be most suitable for embolization in this case. Unintended embolization of nontarget sites due to NBCA migration is a major drawback. However, we avoided this by injecting NBCA under real-time US guidance. Additionally, the concern of adhesion to the embolization device, such as a catheter, which is another drawback of NBCA use, is less likely to occur with injection from a metal needle, as in the present method.

Although the precise etiology of DFAs in the present case is unclear, we considered that they are associated with atherosclerosis because the patient had no clinical, laboratory, or imaging findings indicating systemic vascular disease and vasculitis. As with other peripheral aneurysms, atherosclerotic changes are known to be a common cause of DFA.^2)^ Other causes of DFA include trauma, iatrogenic injury, autoimmune disease, and collagen vascular disease.^1,2,4,8)^

## Conclusion

We reported the case of a DFA aneurysm with a ligated proximal artery that was successfully treated in a less invasive manner by percutaneous direct puncture embolization with NBCA. We propose this method as an alternative treatment option for DFA aneurysm, especially when an antegrade approach is difficult.
